# Category production norms for 117 concrete and abstract categories

**DOI:** 10.3758/s13428-021-01787-z

**Published:** 2022-06-01

**Authors:** Briony Banks, Louise Connell

**Affiliations:** 1 Department of Psychology, Fylde College, Lancaster University, Bailrigg, Lancaster, LA1 4YF UK; 2 Department of Psychology, Maynooth University, Maynooth, Co. Kildare, Ireland

**Keywords:** Category production, Semantic fluency, Categories, Abstract concepts, Concrete concepts

## Abstract

We present a database of category production (aka semantic fluency) norms collected in the UK for 117 categories (67 concrete and 50 abstract). Participants verbally named as many category members as possible within 60 seconds, resulting in a large variety of over 2000 generated member concepts. The norms feature common measures of category production (production frequency, mean ordinal rank, first-rank frequency), as well as response times for all first-named category members, and typicality ratings collected from a separate participant sample. We provide two versions of the dataset: a referential version that groups together responses that relate to the same referent (e.g., *hippo*, *hippopotamus*) and a full version that retains all original responses to enable future lexical analysis. Correlational analyses with previous norms from the USA and UK demonstrate both consistencies and differences in English-language norms over time and between geographical regions. Further exploration of the norms reveals a number of structural and psycholinguistic differences between abstract and concrete categories. The data and analyses will be of use in the fields of cognitive psychology, neuropsychology, psycholinguistics, and cognitive modelling, and to any researchers interested in semantic category structure. All data, including original participant recordings, are available at https://osf.io/jgcu6/.

## Introduction

**Fig. 1:**
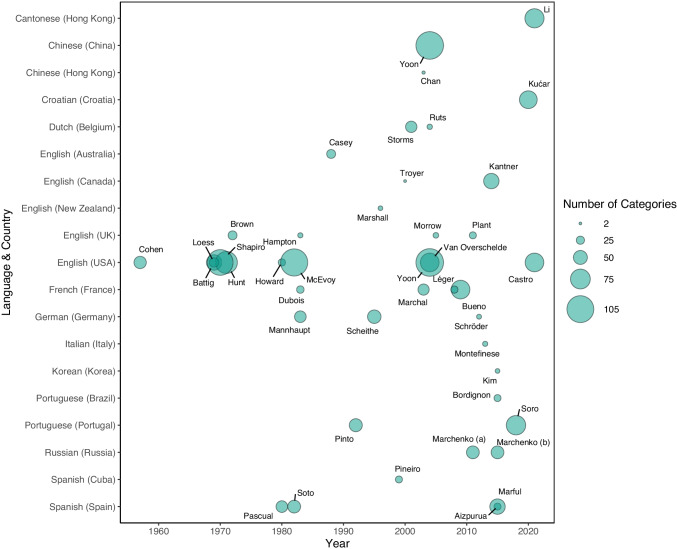
Published category production norms per year, country and number of categories. *Note.* Plotted studies represent normative category production data from adult, non-clinical populations published in a peer-reviewed journal, book, or conference proceedings between 1957 and 2021, not all of which are currently available as datasets. Studies are ordered alphabetically by language and region; circle size indicates the number of categories included in the study, and labels indicate the first author

The ability to categorize concepts is a vital part of human cognition that allows us to understand and interpret the world around us. Accordingly, category production (also termed semantic or verbal fluency) is a widely used task in both cognitive and neuropsychology that is considered to reflect the structure and organization of the conceptual system, and particularly the taxonomy of concepts in semantic memory. A category production task simply requires participants to name concepts that belong to a given category, such as ANIMALS^1^ or EMOTIONS. It is used in a wide range of research, but particularly to investigate underlying categorical and conceptual structure (e.g., Crowe & Prescott, 2003; Hampton & Gardiner, 1983; Rosch, 1975; Troyer, 2000), semantic memory (e.g., Binney et al., 2018; Ryan et al., 2008), and executive function (e.g., Baldo & Shimamura, 1998; Fisk & Sharp, 2004; Shao et al., 2014). The task is also an important tool in clinical research (e.g., Bokat & Goldberg, 2003; Henry & Crawford, 2004) and diagnosis (e.g., Quaranta et al., 2016; Zhao et al., 2013). The importance of the category production task across multiple cognitive domains, and its use in both research and clinical settings, has led to numerous sets of category production norms being published in the last few decades. The first such norms were collected in the USA in 1957 (Cohen et al., “The Connecticut Norms”), and were subsequently updated by Battig and Montague (1969) in their widely cited set of norms. Since then, category production norms have been published in at least nine different languages (see Fig. 1), which have been used in a wide range of psychological research, including psycholinguistics (e.g., Stadthagen-Gonzalez et al., 2017; Warriner et al., 2013), memory (e.g., Ryan et al., 2008; Veling & van Knippenberg, 2004), language comprehension (e.g., Federmeier et al., 2010; Jahncke et al., 2013), cognitive ageing (e.g., Ferreira et al., 2019; Raz et al., 1998), and disorders such as schizophrenia (e.g., Brébian et al., 2010; Vinogradov et al., 1992) and Alzheimer’s disease (McDowd et al., 2011; Ober et al., 1991).

Category production norms have been collected from a variety of geographical regions, spanning North and South America, Europe, Asia, and Australia (see Fig. 1). Nevertheless, most English-language norms to date have been collected in the USA. To our knowledge, only four relatively small sets of category production norms have originated in the UK: Brown (1972; 28 categories), Hampton and Gardiner (1983; 12 categories from the Battig & Montague norms), Morrow and Duffy (2005; 14 categories comparing data from younger and older adults), and Plant et al. (2011; 10 concrete noun and 10 ad hoc verb categories). Thus, a contemporary and comprehensive set of category production norms from the UK is not currently available. Localizing category production norms by region is important because comparisons between norms have found that, while certain categories appear to have minimal differences between geographical regions, many other categories show large geocultural variation (Brown, 1978; Hampton & Gardiner, 1983; Kantner & Lindsay, 2014). For example, Brown (1978) compared norms collected at the same time in Scotland, UK (Brown, 1972) and the United States (Battig & Montague, 1969). Across the 12 categories compared, half had relatively similar patterns of production frequency (CHEMICAL ELEMENTS, UNITS OF TIME, FOUR-FOOTED ANIMALS, COLOURS, MUSICAL INSTRUMENTS, PRECIOUS STONES: Pearson’s *r* = .88 to .61) but some categories differed substantially between locations in their listed category members (OCCUPATIONS OR PROFESSIONS, ARTICLES OF CLOTHING, SPORTS, and BIRDS: *r* = .29 to −.06). Similar patterns were observed by Hampton and Gardiner (1983) when comparing their norms collected in London, UK, with Battig and Montague’s (1969) American norms. Their most frequently named member concepts were very similar for certain categories (e.g., for FRUIT, the top four members in both norms were *apple*, *orange*, *pear* and *banana*), but quite different for others (e.g., for SPORT, the top three member concepts produced by UK participants were *soccer* [*football* in the UK], *rugby*, and *tennis*, but for US participants these were [American] *football*, *baseball*, and *basketball*).

Category structure can also vary over time. Van Overschelde et al. (2004) compared their norms with the Battig and Montague norms from 1969, both of which were collected in the USA: production frequency for the categories COLOURS and PARTS OF THE BODY were highly correlated (*r* > .90; e.g., the top four colours were identical in both studies: *blue*, *red*, *green*, and *yellow*), while the categories A TYPE OF DANCE and A COLLEGE OR UNIVERSITY correlated weakly (*r* = .05 and *r* = .20, respectively), possibly reflecting changing societal and cultural preferences in the 30 years between the studies (e.g., the top four dances in 1969 were *waltz*, *frug*, *twist*, and *foxtrot*, while in 2004 they were *ballet*, *tango*, *salsa*, and *hip hop*). Given the potential variation resulting from chronological and geographical differences in data collection, even within the same language, providing a contemporary set of norms collected in the UK is a timely and important addition to the study of semantic category structure and language.

To date, most category production norms have largely focused on concrete rather than abstract categories. While the Battig and Montague (1969) norms included a relatively diverse range of categories, they were nevertheless predominantly concrete in nature (e.g., VEGETABLE, SHIP, PART OF THE HUMAN BODY, PRECIOUS STONE). As many subsequent studies have simply replicated the Battig and Montague norms either in full or using a smaller subset (e.g., Howard, 1980; Marful et al., 2015; Marshall & Parr, 1996; Storms, 2001), they too have focused on concrete categories, particularly those of historical interest to theories of conceptual processing, such as the basic-level categories outlined by Rosch (1973; e.g., TREE, FRUIT, FISH, BIRD, MUSICAL INSTRUMENT, TOOL, CLOTHING, VEHICLE, and FURNITURE), and the superordinate category ANIMAL. Indeed, category production norms have often focused on just a few high-frequency concrete categories (e.g., Chan et al., 2003; Hampton & Gardiner, 1983; Morrow & Duffy, 2005; Schröder et al., 2012; Troyer, 2000). As a result, far fewer category production norms have been collected for abstract categories; for example, only 15 of the original 56 categories by Battig and Montague could be classed as abstract (e.g., SCIENCE, PART OF SPEECH). These norms were replicated and extended by Van Overschelde et al. (2004) in English, and by Bueno and Megherbi (2009) in French, but only one and three new abstract categories, respectively, were added (e.g., FOOTBALL PENALTY, ACADEMIC DISCIPLINE). A larger number of abstract categories were included by McEvoy and Nelson (1982; 26 abstract categories out of a total of 106, e.g., COLLEGE LEVEL and SEASON) and by Yoon et al. (2004; 26 abstract categories out of a total of 105, e.g., EMOTION and MATHEMATICAL OPERATION). Nevertheless, abstract categories still comprised a relatively small proportion of the data, and neither study purposely selected categories on the basis of abstractness; rather, they were selected based on expected category size (McEvoy & Nelson, 1982) and for the purpose of cross-linguistic comparison (Yoon et al., 2004). To our knowledge, no previous category production norms have explicitly selected a substantial number of abstract categories, or examined differences between them and concrete categories. Thus, less is known about the structure of abstract compared to concrete categories—for example, which member concepts are most frequently named, and the properties of generated concepts such as typicality or, indeed, concreteness. Given the current interest in the cognitive basis of abstract concepts (Borghi et al., 2017; Connell et al., 2018; Desai et al., 2018; Ponari et al., 2020), it seems timely to publish category production norms and comparisons for a larger number of abstract categories alongside more traditional concrete ones.

Certain measures of category production have commonly been used across sets of norms to examine the structure of categories and commonalities in participant responses, particularly how frequently each concept is produced per category (overall, or as the first-produced concept) and in what order (i.e., the ordinal rank of each concept per category). However, other measures may offer important insights into the process of category production; in particular, response times (RT) are a common implicit measure of conceptual processing, and in a category production task would represent the processing time (and indirectly the effort or difficulty) involved in accessing a category member from long-term memory. RT can thus provide insight into how we retrieve particular member concepts from semantic memory and the relationship between the category label and member concepts that come to mind. Indeed, RTs have recently been used to examine the role of sensorimotor similarity and linguistic distributional relations between the category label and the first-produced category member (Banks et al., 2021), as well as neuropsychological deficits (Rohrer et al., 1995) and individual differences (Luo et al., 2010; Shao et al., 2014) in category production. However, only one extant set of category production norms has included RT as a variable: Van Overschelde et al. (2004) reported RTs for all responses given within 30 seconds, but these RTs were measured from the *offset* of typing a response (i.e., pressing an enter key when finished) rather than the *onset*, and so the measured latencies conflate both processing effort to think of a response and typing time to record it. Latencies from verbal responses can provide a more accurate measure of RT, as they can be measured from the exact onset of speech following presentation of the category name. In the present norms, we therefore took the approach of asking participants to generate verbal responses, which enabled us to accurately measure RT for each first-named category member from the onset of speech following stimulus presentation. We report mean response latencies for item-level data (trial-level RTs per participant are provided as supplemental material), which may prove particularly useful in understanding the mechanisms behind generating initial category members (see e.g., Banks et al., 2021).

At this point, it is worth noting two common inconsistencies in the methodologies of category production norms. Firstly, norming procedures often differ in the number of responses participants are allowed to make. Many studies replicate the original method employed by Cohen et al. (1957), which allowed as many responses as possible within 30 seconds, while others have limited the number of responses to just one (McEvoy & Nelson, 1982) or the first few only (Kantner & Lindsay, 2014; Montefinese et al., 2013; Yoon et al., 2004), potentially limiting the usefulness of these datasets as research tools. Allowing participants to generate many category members rather than just a few, and allowing a longer time limit (e.g., 60 seconds) so as to avoid cut-offs for particularly slow or profuse responses, allows the full diversity of category production responses to be recorded; we therefore took this approach in the norms reported here. Such data can potentially allow more in-depth study of category structure, for example semantic clustering (Troyer, 2000; Troyer et al., 1997), or the mechanisms behind activation of concepts from long-term memory (Banks et al., 2021).

Secondly, there are often differences in how studies handle lexical and morphological differences in participant responses, which affects the relevant frequency counts and ranks for member concepts. Category production tasks often generate responses that have the same core referent concept but that vary in their precise word form in terms of morphology (e.g., FRUIT: *apple* vs *apples*; EMOTION: *happy* vs *happiness*) or vocabulary/synonym choice (e.g., FURNITURE: *couch* vs *sofa;* RELATIVE: *dad* vs *father*). Such responses have been handled in a variety of ways in previous norms. Some studies have largely preserved lexical and morphological distinctions apart from minor spelling variations, such as *hi-rise* and *high-rise* (e.g., Howard, 1980; Ruts et al., 2004; Yoon et al., 2004). However, others have grouped morphological variations (e.g., Battig & Montague, 1969; Bueno & Megherbi, 2009; Kantner & Lindsay, 2014; Marchenko et al., 2015; McEvoy & Nelson, 1982; Montefinese et al., 2013; Plant et al., 2011; Van Overschelde et al., 2004) or synonymous responses (e.g., Castro et al., 2021; Marful et al., 2015; Montefinese et al., 2013; Van Overschelde et al., 2004) under one lexical entry. Although any method of grouping responses is inherently subjective, it can be useful for examining broad similarities in semantic category structure. An alternative approach is to provide both grouped and full responses, as in Van Overschelde et al. (2004), allowing for easy comparison with previous norms which have used different data preparation methods, whilst also preserving more fine-grained linguistic and semantic differences. We therefore used this approach in the current study, compiling two sets of our norms: a *referential* version with morphological and synonymous variations (i.e., those referring to the same core referent) grouped together under one label, and a *full* version with all lexical and morphological variations of responses preserved.

Finally, typicality ratings—that is, how good an example of its category is a particular concept—are often included in category production norms alongside measures of frequency and ordinal rank (e.g., Izura et al., 2005; Léger et al., 2008; Montefinese et al., 2013; Plant et al., 2011; Ruts et al., 2004; Schröder et al., 2012). Typicality has frequently been studied as a measure of graded category structure (e.g., Osherson & Smith, 1981; Rosch, 1975; Rosch et al., 1976), and can predict the frequency and rank order of category production responses (e.g., Hampton & Gardiner, 1983; Mervis et al., 1976; Montefinese et al., 2013; Uyeda & Mandler, 1980). In addition to our category production data, we thus include typicality ratings for the majority of category–member pairs from a separate sample of participants.

### The present study

To summarize, we present a large set of category production norms that has several advantages over existing norms in the English language. First, to the best of the authors’ knowledge, they comprise the largest and most contemporary set of category production norms collected in the UK. Second, they comprise production norms for the largest number of concrete and abstract categories in English to date: 67 concrete and 50 abstract categories (117 in total). Given the large number of categories, the norms span a variety of category levels and subtypes: subordinate, basic, and superordinate taxonomic levels, as well as semantic categorical divisions such as natural and artefact, animate and inanimate, social and non-social category types. Third, we provide two versions of our category production norms: a referential version (which groups responses with the same referent, and forms the basis of the analyses reported here) and a full version (which leaves each response in its original word form, with analyses included as supplemental material). Researchers may select the most appropriate version for their required purpose. Fourth, by allowing participants to provide as many responses as possible within 60 seconds, we have generated a very large and comprehensive dataset for future research, containing 2445 unique category–member pairs in the referential version (5475 including idiosyncratic items produced by only one participant), or 2557 unique pairs in the full version (6448 including idiosyncratic items). Fifth, we include RTs for all first-named concepts per category. Although we do not report RTs for every response, the majority of audio recordings (those where participants gave permission) are available for other researchers to calculate such timings if desired. Sixth, and finally, we provide typicality ratings for 2234 member concepts within their categories (87% of items in the full version; 80% of items in the referential version), collected from a separate sample of participants, to enable analysis of categorical gradedness.

To validate the norms, we present an analytical comparison between the present data and previous sets of English-language norms, from both the UK (Hampton & Gardiner, 1983) and the USA (Van Overschelde et al., 2004). We also report a number of structural and psycholinguistic differences between abstract and concrete categories, which highlights the importance of making available category production norms for abstract as well as concrete categories. These norms have already proven useful in our own lab, where we have used them to study and computationally model the process of conceptual activation during category production (Banks et al., 2021). We hope that the norms will be of interest and use to researchers in a broad range of cognitive and psychological research, and any field seeking to gain insight into the processes involved in selecting and retrieving category members from semantic memory.

## Study 1: Category production norms

### Methods: Category production norming

#### Participants

Sixty-four participants recruited from Lancaster University took part for payment of £3.50 GBP. Participants were recruited from the general student and staff population of Lancaster University, and likely included a proportion of Psychology undergraduates, although we did not collect details of course subject or academic background as part of our demographic data. Three participants were excluded as they were non-native speakers of English (i.e., questioning during debriefing revealed that they had misunderstood the screening criteria), and one was excluded for providing too few responses (*M* < 2 responses per category). Of the remaining 60 participants, all had English as their native language, 46 were female, mean age was 21.72 years (*SD* = 5.73), and 52 were right-handed. The study received ethical approval from the Lancaster University Faculty of Science and Technology Research Ethics Committee. Participants gave their informed consent to take part and to publicly share their anonymized data, and could additionally opt in to sharing publicly their original voice recordings with anonymized filenames: 52 out of 60 participants consented to do so.

#### Materials

We selected 117 categories representing a range of concrete and abstract concepts (see Table 1), the majority of which were selected from the categorization literature (Battig & Montague, 1969; Capitani et al., 2003; Larochelle et al., 2000; McEvoy & Nelson, 1982; Rosch, 1975; Uyeda & Mandler, 1980; Van Overschelde et al., 2004). Where possible, categories spanned multiple taxonomic levels, such as the basic (e.g. BIRD), superordinate (e.g. ANIMAL), and subordinate (e.g. WATER BIRD) levels. The 67 concrete categories represented a range of living and non-living, animate and inanimate, artefact and natural, and biological and non-biological semantic categories. We included many common concrete categories that have been frequently investigated in the categorization literature (e.g., FRUIT, MUSICAL INSTRUMENT), as well as other less common concrete categories (e.g., BIRD OF PREY, ROOM IN A HOUSE). The 50 abstract categories covered social and non-social, human and non-human, and internal (i.e., relating to internal human experience) and external semantic categories. Some of these categories had been previously included in category production norms (e.g., SCIENCE, EMOTION) while others were novel to the present study. Some of the novel abstract categories were subordinate (e.g., VIOLENT CRIME, NEGATIVE EMOTION) or modified variants of those already selected from the literature (e.g., ROYAL TITLE), while others were created de novo by the authors based on categorical distinctions in WordNet (Princeton University, 2010) for abstract entities (e.g., PERSONAL QUALITY, FRACTION, SOCIAL GATHERING). All categories were piloted on participants not involved in the main study to ensure that they were understandable. Categories were divided into three lists of 39 categories each, counterbalanced as much as possible across the abstract/concrete dimension. Categories that constituted a subset of another category (e.g., WATER BIRD, BIRD) were not included in the same stimulus list. Four additional categories (BREAD, CIRCUS ACT, FOOTWEAR, and CONTINENT) that were not featured in the main task were used as practice items to ensure participants had understood the instructions.

**Table 1:** All 117 categories featured in the category production norms, comprising 50 abstract and 67 concrete categories

Abstract categories			
Academic subject	Injury	Profession	Team sport
Art form	Legal profession	Psychological illness	Three-dimensional shape
Artistic movement	Medical specialty	Racket sport	Time of day
Book genre	Military title	Religion	Two dimensional shape
Crime	Month	Royal title	Type of word
Day of the week	Negative emotion	Science	Unit of length
Disease	Negative personal quality	Season	Unit of time
Emotion	Non-violent crime	Social gathering	Unit of weight
Family relationship	Personal quality	Social relationship	Violent crime
Fraction	Political system	Sport	Water sport
Geometric shape	Positive emotion	Statistical term	Winter sport
Healthcare profession	Positive personal quality	Supernatural being	
Infectious disease	Prime number	Symptom of illness	
Concrete categories			
Alcoholic drink	Dairy product	Human dwelling	Religious building
Animal	Drug	Insect	Rodent
Bathroom fixture	Fabric	Jewellery	Room in a house
Bird	Farm animal	Kitchen appliance	Snake
Bird of prey	Fish	Kitchen utensil	Spice
Boat	Flower	Living room furniture	Stinging insect
Body of water	Four-legged animal	Meat	String instrument
Breed of dog	Four-wheeled vehicle	Metal	Tool
Building	Fruit	Musical instrument	Tree
Building material	Fuel	Natural landform	Two-wheeled vehicle
Camping equipment	Furniture	Nut	Vegetable
Carpenter's tool	Gardening tool	Part of a boat	Vehicle
Chemical element	Gemstone	Part of a building	Water bird
Citrus fruit	Green vegetable	Part of a tree	Weapon
Clothing	Hair colour	Part of the body	Weather
Colour	Hat	Part of the face	Wind instrument
Cosmetic	Herb	Reading material	

#### Procedure

Following consent procedures, participants sat individually in front of a computer screen while wearing a headset microphone. They read instructions that asked them to name aloud as many concepts as possible that belonged to each category, within a maximum of 60 seconds (exact task instructions are provided as supplemental materials on the OSF). PsychoPy (version 1.85.4) was used to present the stimuli and audio-record all responses. Participants triggered the start of each trial by pressing the space bar on the keyboard. Each trial began with a fixation cross for 500 ms followed by the category name presented in capital letters in the centre of the screen. The category name remained onscreen until participants could not name any more concepts and pressed the spacebar to end the trial, or until the trial timed out automatically after 60 seconds. When a trial ended, the words “Press space bar when ready” then appeared onscreen until participants triggered the next trial; timing between categories was thus self-paced, and participants could take a short break between categories if required. Participants first carried out the four practice trials, and were then randomly assigned to one of the three category lists. Each list was presented to 20 participants, and categories from each list were presented in randomized order for each participant. Verbal responses were audio-recorded through a headset microphone and were simultaneously transcribed during the task by the experimenter (hidden from the participant’s view behind a panel screen); these transcriptions were later verified via the audio recordings. Unintelligible responses (comprising 0.08% of all responses) were coded as such and are represented as skipped ranks in the trial-level dataset. The entire experimental procedure took approximately 20 minutes, after which participants provided demographic information and were debriefed by the experimenter.

#### Norms data preparation

In preparing the norming data, we took a bottom-up, data-driven perspective on category membership that included any concepts that our participants considered to belong to a given category (see similar approaches in Battig & Montague, 1969; Van Overschelde et al., 2004). That is, we did not apply a top-down, constraint-driven perspective by selecting category members for inclusion based on whether they might be considered “true” or “correct” members of that category, but future researchers can apply such constraints as appropriate for their particular research.

##### Full version

All transcribed responses were included in this dataset exactly as they were spoken, preserving morphological differences such as agreement and grammatical tense, and using British English spellings.

##### Referential version

For each category, responses which referred to the same core referent were combined under one grouping label: specifically, the response most frequently produced by participants. This referential grouping applied to any morphological variations (e.g., singular and plural forms of a word such as ANIMAL: *cheetah/cheetahs* → *cheetah*; different parts of speech such as EMOTION: *happy*/*happiness* → *happy*), and synonymous variations (e.g., FAMILY RELATIONSHIP: *mum/mother* → *mother*) in the terminology used to label a referent. Where an equal number of responses were produced for each variant label (e.g., an equal number of participants named *rucksack* and *backpack* for the category CAMPING EQUIPMENT), we selected the word with the higher frequency in British English as the grouping label (e.g., rucksack/backpack → *rucksack*) based on frequency counts for unigrams and bigrams from the SUBTLEX-UK corpus (van Heuven et al., 2014). If neither variant label appeared in the SUBTLEX-UK corpus (e.g., *sandwich maker* and *sandwich toaster* for the category KITCHEN APPLIANCE), we carried out a search on British English texts from 1999–2019 in the Google Books Ngram Viewer (http:// books.google.com/ngrams), and selected the word with the higher frequency count as the grouping label (e.g., *sandwich maker*); 20 items were selected for use this way. In three cases, an equal number of participants produced full (two-word) and abbreviated (single-word) variants of a response (e.g., CARPENTER’S TOOL: *spirit level/level*), where the single-word term was polysemous and therefore frequency counts would be inaccurate; for these cases, the unabbreviated version was selected as the grouping label to avoid ambiguity. Responses that were closely related but did *not* refer to the same core referent were maintained as separate items, such as subordinate categorical distinctions (e.g., *wine* and *white wine*).

##### Category production measures

We calculated three measures of category production at the category level: *category size* was the total number of unique, non-idiosyncratic member concepts (i.e., concepts produced by more than one participant) that were listed for a given category across the entire set of participants; *mean number of responses* was the average number of responses produced by each participant for a given category, calculated as the total number of non-idiosyncratic responses collated for that category divided by the number of participants who saw that category. We also calculated a novel measure of *category openness* to distinguish between closed categories (i.e., where each participant named the same fixed set of category members) and more open-ended categories (where each participant tended to name completely different category members). The measure was calculated as *openness* = 1 − (*mean number of responses* / *category size*), where 0 reflects a completely closed category and 1 reflects a completely open category.

At the item level, we calculated several measures of category production: *production frequency* (the number of participants who named a particular member concept within its category); *first-rank frequency* (the number of participants who named a particular member concept *first* within its category, where responses that were never named as a first response by any participant were excluded rather than given a frequency of zero); and *mean rank* (the mean ordinal position of a particular member concept within its category). We also calculated *weighted rank:* a modified Borda count for open-ended responding based on the maximum rank in the dataset (i.e., 32), whereby the production frequency of each member concept in its category was weighted by the ordinal rank position in which each individual participant named it. For each participant and category, first responses were scored as 32, second responses as 31, third responses as 30, and so on. Weighted rank is the sum of these scores per category–member item, where higher values indicate category members produced both early and often and lower values indicating category members produced rarely and/or later in response lists. Finally, we calculated the mean RT for first-named member concepts within their category. RT per trial was measured from the onset of the category name until onset of speech to name the first member concept (dysfluencies were disregarded). RT onset was determined by PsychoPy from the onset of the category name, and RT offset was measured by experimenter markup of speech onset in Praat (Boersma & Weenink, 2018); notwithstanding human error in the latter, we estimate RT measurement error to be within ±1 ms^2^.

The 22 contains a full list of variables featured in the norms; all measures were calculated separately for the full and referential versions. Repeated responses in both versions of the norms were disregarded and did not contribute to the calculation of any category production measures. Summary statistics and Spearman’s correlations between all measures of category production were calculated in JASP version 0.14.1 (JASP Team, 2020).

### Methods: Typicality ratings

#### Participants

In order to recruit a sample with similar linguistic experience to the category production study, we restricted recruitment to native speakers of English who were UK nationals on the online research crowdsourcing tool Prolific. A total of 141 native speakers of English took part in this study via Prolific; however, 14 participants’ submissions were rejected because their ratings did not pass our quality control checks (i.e., they were not paid and their data were excluded; see *Data Preparation* below). New participants were recruited via Prolific until we reached *N* = 12 for all stimulus lists (participants who were not rejected were able to rate multiple lists). A total of 127 participants were included in the final analysis (88 female, mean age = 31.23 years, *SD* = 10.33 years, 111 right-handed) and received £1.75 GBP for participation. Ethical approval was gained from the Lancaster University Faculty of Science and Technology Research Ethics Committee, and all participants gave their informed consent to take part and openly share their anonymized data.

#### Materials

Stimuli for typicality ratings comprised 2280 category–member word pairs: 2234 pairs from the full version of the present norms (this dataset comprised all items used in the analysis of Banks et al., 2021), plus an additional 46 pairs that were rated for use in a separate study and do not feature in the present norms. Category–member pairs were pseudo-randomly divided into 20 stimuli lists, whereby each category was distributed across lists as equally as possible. Member concepts that appeared with more than one category (e.g., *eagle* for the categories BIRD and BIRD OF PREY) or that appeared in both singular and plural forms (e.g., *apple* and *apples* for the category FRUIT) were allocated to separate lists. In addition, production frequency (see *Measures of category production*) and log word frequency (LgSUBTLWF, from the English Lexicon Project, Balota et al., 2007) were counterbalanced across lists (mean production frequency per list = 5.73 [SD = 4.65] ranging from 5.24 to 6.05, with no significant difference between lists, *F*[19] = 0.28, *p* = 0.999; mean LgSUBTLWF per list = 2.59 [SD = 0.87] ranging from 2.48 to 2.74, with no significant difference between lists, *F*[19] = 0.98, *p* = 0.485).

We selected 80 items from our stimuli with known typicality ratings from previous studies (Armstrong et al., 1983; Rosch, 1975; Uyeda & Mandler, 1980) to use as quality control checks in online data collection. Half of these had high typicality ratings (i.e., < 1.6 on a scale of 1–7, 1 being high typicality and 7 being low typicality; *M* = 1.34, *SD* = 0.19) and half had low typicality ratings (i.e., > 3.7, *M* = 4.39, *SD* = 0.54); each stimulus list contained four high-typicality and four low-typicality control items. Two further items *not* featured in our stimuli were used as scale calibrators: one high typicality (TOY: *doll*) and one low typicality (VEHICLE: *surfboard*), presented at the start of each stimulus list; these items are not included in the present norms. Each stimulus list therefore comprised 120 items (category–member word pairs), including the two calibrator and eight control items.

#### Procedure

Each stimulus list was presented in randomized order in an online questionnaire via Qualtrics. For each item, the category name was presented in capital letters in the centre of the screen, above the framing question “How good an example of this category is/are a/an X(s)?” (e.g., ANIMAL: “How good an example of this category is a *cat*?”) and the rating scale 1–5 (with 1 being a “very poor” example, and 5 being a “very good” example). Participants were asked to base their ratings on their own judgements (exact task instructions are provided as supplemental material on the OSF). Participants responded using a mouse, where only one response per item was allowed, but participants could also indicate if they did not know the meaning of the category or the category member (no ratings were recorded for such trials). The entire ratings procedure took approximately 15 minutes. At the end of the stimulus list, participants provided demographic information and read a study debrief.

#### Typicality data preparation

To check the quality of the online data, each participant’s ratings for the control items were correlated with ratings gained from previous studies. If the Pearson’s correlation coefficient was *r* < .30, *and* the variance of that participant’s data was close to zero, then the participant was excluded for failing to adequately attend to and/or understand the task. Fourteen participants were excluded on this basis (see Participants). We calculated inter-rater reliability for each stimulus list using Cronbach’s alpha calculated using the psych package in R (Revelle, 2021). Inter-rater reliability was high for each stimulus list (*r* ≥ .79 for all lists, range .79 to .90). For each category–member pair, we then calculated the mean typicality rating across all participants. These item-level ratings are provided in the full category production dataset. For the referential version of the data, where responses were grouped according to their core referent, the mean typicality rating was used for grouped items (e.g., for the category–member pair ANIMAL: *cheetah*, both singular and plural responses were grouped together; thus the mean typicality rating for *cheetah* and *cheetahs* was used). Correlations between typicality ratings and measures of category production were calculated and plotted using the ggcorr function from the GGally package (Schloerke et al., 2021) in RStudio version 1.3.959. As the measures were differentially distributed, which can artificially restrict the value of Pearson’s correlation (J. Cohen et al., 2013), we opted to calculate Spearman’s correlation as the measure of association between variables.

### Results

Summary statistics across all categories for both full and referential versions of the norms are shown in Table 2. Analyses of the norms reported in this section focus on the referential version of the data, although analyses of the full version are also provided as supplemental material at https://osf.io/jgcu6/. Idiosyncratic items (i.e., category members named by only one participant; also provided as supplementary material) were excluded from all analyses, resulting in a total of 2445 distinct category–member pairs for the referential dataset at the item level. As the analyses reported here were exploratory, no inferential statistics are reported.

**Table 2:** Summary statistics for category production measures across all 117 categories (both versions of norms, excluding idiosyncratic items), with total number of items for each measure and the mean and standard deviation

Variable	Referential version			Full version		
	*N*	Mean	SD	*N*	Mean	SD
Category level						
Category size	2445	20.90	10.37	2558	21.86	11.08
Mean number of responses	14639	6.45	3.04	13817	6.10	2.94
Category openness	14639	0.67	0.13	13817	0.70	0.13
Item level						
Production frequency	2445	5.97	4.92	2558	5.38	4.43
Mean rank	2445	6.43	3.68	2558	6.22	3.67
Weighted rank	2445	164.42	147.41	2557	149.18	133.14
First-rank frequency	675	3.16	3.60	722	2.88	3.21
RT (seconds) first response	675	3.65	2.12	722	3.56	2.08
Typicality rating	1956	4.18	0.67	2234	4.21	0.66

The *N* for *mean number of responses* is larger in the referential version due to the grouping of similar concepts and thus fewer idiosyncratic responses being excluded than in the full version

**Fig. 2:**
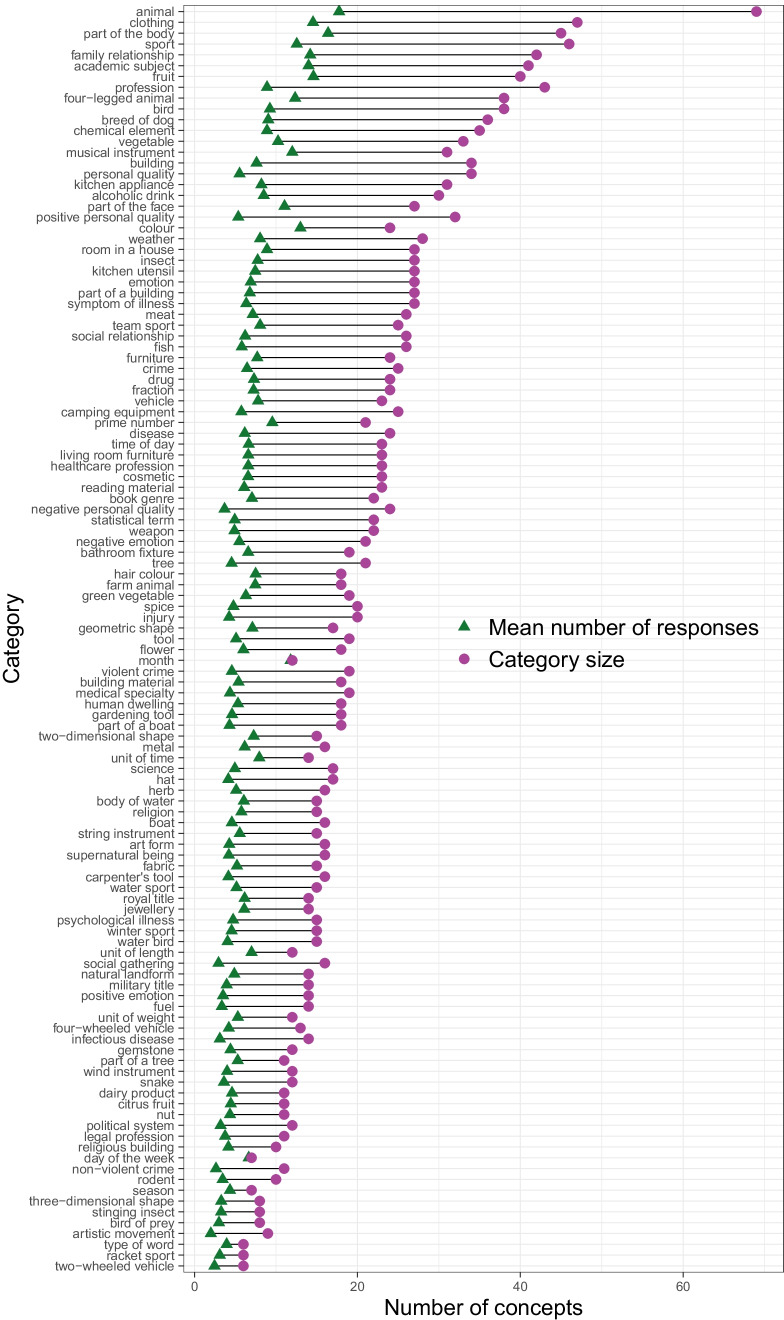
Lollipop plot of category size and mean number of responses per category

At the category level, Fig. 2 shows category size (i.e., total number of unique member concepts) and mean number of responses (i.e., number of member concepts listed by an average participant) for each category. Category size ranged from a very small set of six member concepts (TWO-WHEELED VEHICLE, RACKET SPORT, and TYPE OF WORD) to a very large set of 69 (ANIMAL). Participants named on average 6.47 concepts per category, but this number was highly variable and ranged from 2.00 concepts (ARTISTIC MOVEMENT) to 17.85 concepts (ANIMAL). For certain bounded categories, the category size and mean number of responses were very similar (i.e., MONTH and DAY OF THE WEEK), indicating that participants tended to consistently name the full set of possible member concepts; indeed, these categories had the lowest openness scores of all categories (.02 and .05 respectively; e.g., almost all participants named all seven days of the week). For other categories, responses were more variable; for example, ANIMAL, EMOTION, and TREE have large differences between the total category size and mean number of responses, and accordingly, large openness scores (ANIMAL and EMOTION = .74, TREE = .78) indicating greater inter-participant variability in the subset of member concepts each individual produced for that category. For instance, although participants listed on average seven different types of EMOTION, it represented only 26% of the total category size of 27 members. Openness ranged from .02 (MONTH) to .85 (NEGATIVE PERSONAL QUALITY), although the majority of categories were relatively open, with 93% scoring > .50—that is, individual responses within most categories varied somewhat between participants. Openness was moderately and positively correlated with category size (*ρ* = .45, i.e., larger categories were more open), but was only weakly and negatively correlated with mean number of responses (*ρ* = −.16, i.e., participants tended to give fewer responses to more open categories). Openness was also strongly related to the number of idiosyncratic responses per category (*ρ* = .75), whereby open categories contained more idiosyncratic category members.

**Fig. 3:**
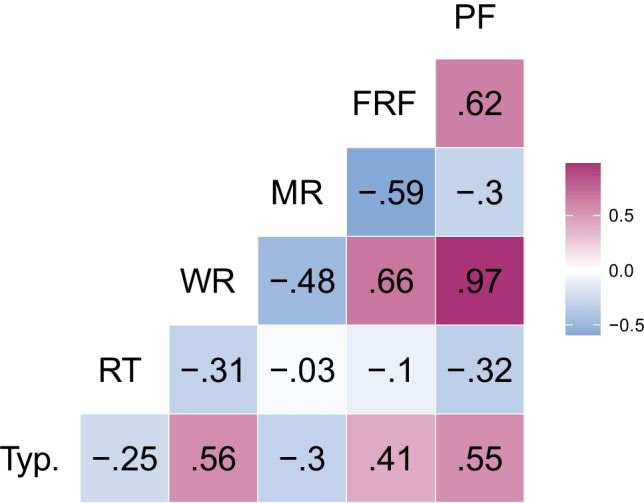
Correlation heatmap (Spearman’s correlations) between measures of category production and typicality. *Note*. PF = production frequency; FRF = first-rank frequency; MR = mean rank; WR = weighted rank; RT = mean first-response time; Typ. = typicality rating

At the item level, we carried out Spearman’s correlations between all five measures of category production and typicality (see Fig. 3). Both production frequency and first-rank frequency were moderately negatively correlated with mean ordinal rank, indicating that more frequent responses were named earlier in the task. Weighted rank was very strongly positively correlated with production frequency, and hence shows a very similar pattern of intercorrelation with other variables. First-response RTs were negatively correlated with production frequency (more frequently produced responses were named faster) and weighted rank (early, frequently produced responses were named faster), but were only very weakly correlated with first-rank frequency and not at all with mean rank. Typicality ratings were moderately correlated with both frequency measures as well as weighted rank (concepts with higher typicality ratings were named more frequently overall and as a first or early response), but were more weakly and negatively correlated with mean rank and RT (more typical responses were named earlier and faster).

### Comparisons with previous norms

We compared our current norms with two previous sets of category production norms: Van Overschelde et al. (2004), a contemporary replication of the Battig and Montague norms collected in the USA; and Hampton and Gardiner (1983), an older set of norms collected in the UK for 12 categories. The goal of this comparison was to allow us to analyse differences in category production across geographical regions but within a relatively similar time frame (i.e., twenty-first-century norms from the UK versus USA), and across time periods but within the same region (i.e., UK norms from late 2010s versus early 1980s). Van Overschelde et al. (2004) gained their norms from at least 600 participants per category (*M* = 672 participants, range = 633–710), while Hampton and Gardiner (1983) had a sample size of 72 participants for all categories. As not all categories in our norms overlapped with those of the other two studies, we analysed only the 44 matching categories from Van Overschelde et al. and the 11 matching categories from Hampton and Gardiner. Several category names in Van Overschelde’s norms were slightly different from our category names, sometimes due to what appeared to be dialectal differences; in such cases, we matched the categories where we judged that they referred to the same semantic class (e.g., ALCOHOLIC BEVERAGE and ALCOHOLIC DRINK; RELATIVE and FAMILY RELATIONSHIP). Because the majority of responses were reported in the singular form by Overschelde et al. and Hampton and Gardiner, we used the singular form of all items in our dataset for the purpose of comparison (e.g., for PART OF THE BODY, we used *hand* instead of *hands*). Minor differences in spelling were also standardized across the three datasets as British English spelling (e.g., *meter* → *metre*; *chicken pox* → *chickenpox*; *sulfur* → *sulphur*), and repetitions of category names were added for consistency (e.g., for the category TREE, the response *apple* was consistently rendered as *apple tree*). Idiosyncratic items were excluded from all norms before comparison; Van Overschelde et al.’s norms already excluded responses produced by < 5% of participants.

All three measures of category production (production frequency, mean rank, and first-rank frequency) were available for comparison in Van Overschelde et al.’s norms, but only production frequency and first-rank frequency were available in Hampton and Gardiner’s norms. As the variables being compared were differentially distributed, as before we opted to calculate Spearman’s correlation as the measure of association between variables (calculated in RStudio version 1.3.959 using the dplyr package: Wickham et al., 2021); these were first calculated globally (i.e., based on all category–member pairs) and then per category. To capture differences in the overlap between responses (i.e., to what extent particular responses were given in one study but not in another), we ran correlations of production frequency on category members produced by participants in *either* relevant study, where absent category members were allocated a production frequency of zero (e.g., the item BIRD: *swan* had a production frequency of 5 in our norms, but was absent from Van Overschelde’s and so received a value of 0). For first-rank frequency and mean rank, correlations were based only on items produced in *both* relevant studies (following Brown, 1978; Kantner & Lindsay, 2014). Note that there were insufficient data to calculate per-category correlations for first-rank frequency (e.g., many categories had 0–2 overlapping first-named members), and so we report global correlations only.

#### Cross-region comparisons

Overall, the present UK norms showed a variable resemblance to the Van Overschelde et al. (2004) USA norms. The global correlation for production frequency was moderate at best (*ρ* = .35, *N =* 1376) while correlations for mean rank (*ρ* = .75, *N =* 595) and first-rank frequency (*ρ* = .63, *N =* 201) were much higher. This pattern is largely due to a relatively low overlap in produced items for certain categories (i.e., many items produced in one set of norms were not produced in the other), but matching items between norms were produced in a similar order and at similar first-response frequency; indeed, when only *matching* items between studies were analysed, the correlation for production frequency was much higher (*ρ* = .72). The difference in correlations underscores that, when comparing category production norms, it is important to consider items that are present only in one dataset and absent in the other because focusing only on overlapping items can inflate the apparent congruence.

**Fig. 4:**
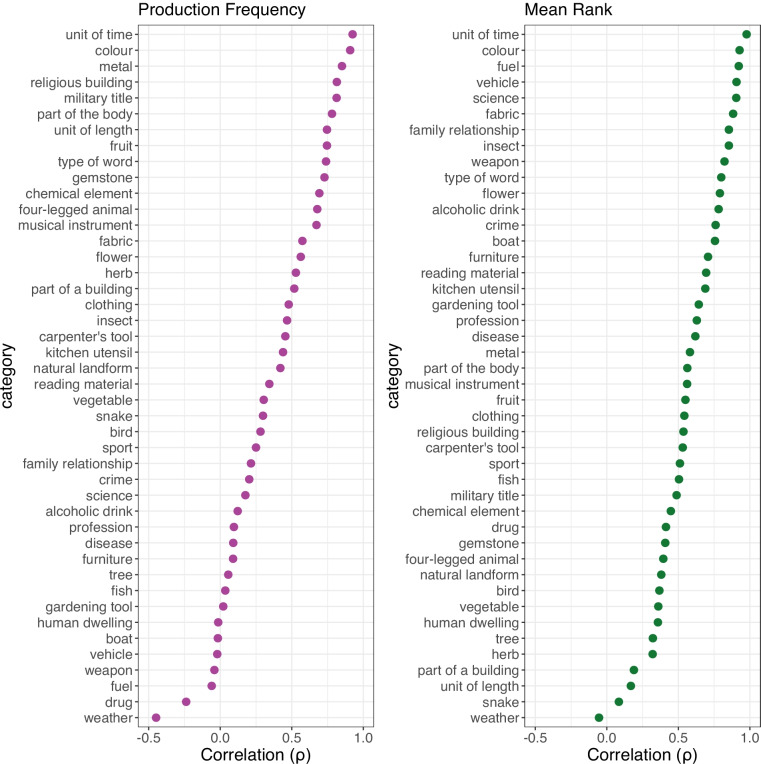
Dot plots of the cross-region comparison between current category production norms (UK) and norms from Van Overschelde et al. (2004: USA), showing per-category Spearman’s correlations for production frequency and mean rank. *Note:* Categories in each plot are ordered by size of correlation coefficient (high to low). Correlations for production frequency include items produced in either study; correlations for mean rank only include matching items produced in both studies

Correlations for individual categories showed large geographic variation (see Fig. 4). While certain categories are very similar between the UK and USA (e.g., UNIT OF TIME, COLOUR, TYPE OF WORD), others greatly differ (e.g., WEATHER, VEHICLE, TREE). These differences appear to be driven by three main factors. Firstly, differences in the natural environment meant that many biological categories had quite different member concepts per region (e.g., half of US participants’ responses to the category SNAKE are species native to North America but not Europe, and were never named by our UK participants). Secondly, but distinctly from the first point, cultural differences had a similar effect on some social and artefact categories (e.g., for ALCOHOLIC DRINK, 45% of UK participants produced *cider* compared to zero US participants^3^, and out of a total of 40 responses across both studies, only nine were produced in both—e.g., *vodka, whiskey*, and *beer*). Lastly, as we did not attempt to control for dialect, differences in terminology were also responsible for some differences in listed category members (e.g., the most frequent responses for the category FUEL were *petrol* in the UK and *gasoline* in the US norms, which in fact have the same referent of refined petroleum). These cross-region patterns closely match previous UK–US comparisons (Brown, 1978; Hampton & Gardiner, 1983), where certain categories were found to be highly consistent between regions (e.g., CHEMICAL ELEMENT, UNIT OF TIME, COLOUR, PRECIOUS STONE, FRUIT—all of which were also highly correlated in the present analysis), and others much less so (e.g., CLOTHING, SPORT, FISH—again, matching the present pattern of results). Critically, the differences we observe here between contemporary category production in the UK versus USA highlight the importance of using geographically appropriate norms in psychological research.

#### Cross-time comparisons

The present norms (collected in 2017–2018) also showed a variable resemblance to the Hampton and Gardiner (1983) norms, collected more than 35 years earlier in the UK. The global correlation for production frequency was moderate (*ρ* = .45, *N* = 584; although, as for cross-region comparisons, when only matching items were analysed the correlation was stronger, *ρ* = .64, *N* = 267), as was that for first-rank frequency (*ρ* = .43, *N* = 54), which suggests that there was limited overlap in the member concepts produced for each category as well as some differences in which items were named first.

**Fig. 5:**
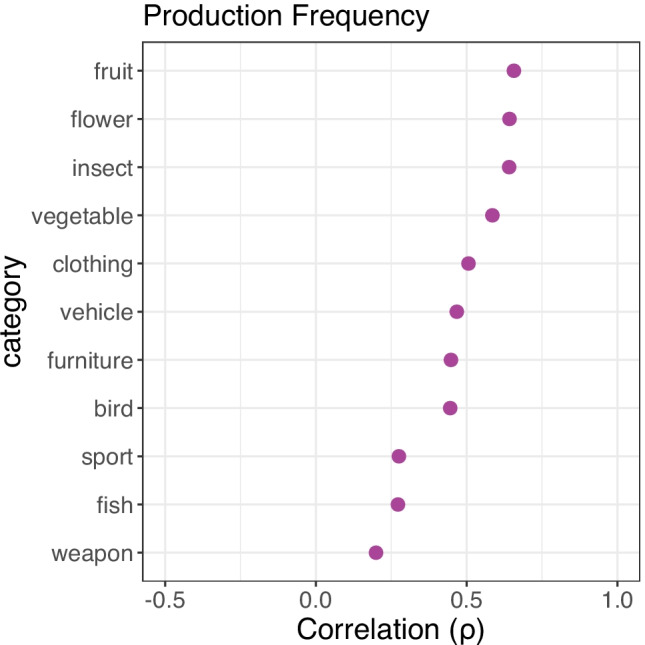
Dot plot of the cross-time comparison between current category production norms (UK) and norms from Hampton and Gardiner (1983: UK), showing per-category Spearman’s correlations for production frequency

Per-category correlations for production frequency were moderate to low (see Fig. 5). Category production responses were somewhat consistent between Hampton and Gardiner’s (1980s) sample and the present (2010s) UK sample for natural categories such as FRUIT, INSECT, FLOWER, and VEGETABLE, but were far less so for others such as WEAPON, FISH, BIRD, and SPORT. Cultural changes can potentially explain many of these differences. For example, in the category CLOTHING, *hoodie* was named by 45% of participants in our norms but was never given as a response in the 1983 norms. Similarly, *basketball* and *running* are both frequent responses for the category SPORT in our norms (named by 80% and 45% of participants, respectively) but were only named by 26% of participants each in the 1980s. Even natural categories can potentially capture cultural shifts, such as differences in the category FISH which likely reflect changes in UK fish-eating habits: *trout* and *plaice* were named by > 50% of participants in 1983, but were named by only 15% of participants in the current data, while *tuna* is presently the most popular response (named by 60% of participants in our norms compared to 19% in 1983). Similar changes over time were observed by Van Overschelde et al. (2004) in the USA, where the largest shifts occurred in culturally dependent categories like TYPE OF MUSIC or TYPE OF DANCE but also in less obvious categories such as CLOTHING and VEGETABLES.

Overall, the cross-region and cross-time comparisons highlight the importance of geographically relevant and up-to-date norms, as we observed many differences between geographic regions and time periods. Nevertheless, some categories such as COLOUR or UNIT OF TIME did show a strong level of robustness across geographical regions, which we speculate is because participants’ experiences of these categories’ member concepts were quite similar. For example, the same colours, called by the same names, tend to occur in similar distributions in both the USA and UK, and hence the category COLOUR was relatively robust across these regions. However, none of the categories available for comparison across time showed such a high degree of robustness, which may be due to the specific categories in question. That is, if categories such as COLOUR had been normed in the 1980s UK study, we may have seen the same pattern of responses as in 2010s UK.

## Study 2: Concrete versus abstract categories

As the present category production norms are the first to collect data for such a wide variety of abstract categories, our goal in this second study is to explore and present what differences exist between abstract and concrete categories in category production behaviour. To this end, we first compare the domains in terms of the measures of category production we outlined in Study 1, at both the item and category level. Furthermore, because abstract and concrete domains often differ in several psycholinguistic variables (i.e., abstract words tend to be longer, of lower frequency, and acquired later, and of course have lower concreteness than concrete words: e.g., Gilhooly & Logie, 1980; Kousta et al., 2011), we also examine whether and how the member concepts of abstract and concrete categories differ in these terms.

### Method

We examined category- and item-level variables for all 2445 member concepts in the referential version of our category production norms, separately for the 67 concrete and 50 abstract categories. As in Study 1, idiosyncratic items were excluded from analysis. In addition, we examined how member concepts for abstract and concrete categories compared across four additional psycholinguistic variables: word frequency (Zipf scores from the SUBTLEX-UK database: van Heuven et al., 2014), word length (calculated using the Stringi package, Gagolewski, 2020, in RStudio version 1.3.959), age of acquisition (Kuperman et al., 2012), and concreteness ratings (Brysbaert et al. (2014). Coverage for the three variables differed: word frequencies were available for 2095 (86%) items, word length for all (100%) items, age of acquisition ratings for 1760 (72%) items, and concreteness ratings for 1892 (77%) items.

### Results and Discussion

Table 3 shows summary statistics for all variables across concrete and abstract categories. Overall, concrete categories were larger in size than abstract categories, containing on average 2.5 more member concepts per category (see Fig. 6). The largest concrete category was ANIMAL, at 69 member concepts, but this category was something of an outlier in its size (see Fig. 2). The next-largest concrete categories were CLOTHING (47 members) and PART OF THE BODY (45 members), which were comparable to the largest abstract categories of SPORT (46 members) and PROFESSION (43 members). Following the same pattern, participants tended to produce slightly more member concepts for concrete than abstract categories, but the mean difference was less than one member per category. Concrete categories were on average slightly more open than abstract, with a narrower distribution (see Fig. 6); participants were more likely to name a different set of category members for concrete categories (e.g., TREE, WEAPON) than for abstract categories (e.g., EMOTION, FRACTION), where participants tended to name relatively similar sets of category members. However, the most open and closed categories (i.e., categories at both tail-ends of the distribution) all tended to be abstract: for instance, PERSONAL QUALITY, PROFESSION, and INJURY were all highly open categories, while TYPE OF WORD, DAY OF THE WEEK, and MONTH were all quite closed.

**Table 3:** Summary statistics for category production measures and psycholinguistic variables in concrete and abstract categories (referential version of norms, excluding idiosyncratic items), with total number of items for each measure and the mean and standard deviation

Variable	Abstract categories			Concrete categories		
	*N*	Mean	SD	*N*	Mean	SD
Category level						
Category size	970	19.40	9.60	1475	22.02	10.85
Mean number of responses	5759	5.95	2.73	8880	6.82	3.23
Category openness	5759	0.65	0.18	8880	0.68	0.07
Item level						
Production frequency	970	5.92	5.16	1475	6.00	4.76
Mean rank	970	6.01	3.57	1475	6.71	3.74
First-rank frequency	276	3.23	3.78	399	3.11	3.47
RT (seconds) first response	276	4.14	2.62	399	3.31	1.62
Typicality rating	734	4.26	0.59	1222	4.13	0.71
Psycholinguistic variables						
Word frequency	813	3.86	0.92	1282	3.80	0.78
Word length	970	7.99	3.12	1475	6.67	2.56
Age of acquisition	655	8.07	2.99	1105	7.09	2.83
Concreteness	719	3.48	0.98	1173	4.69	0.38

**Fig. 6:**
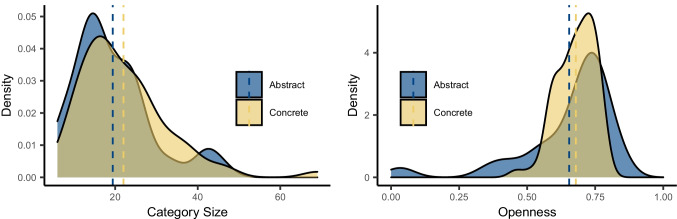
Density plots of category size and openness for abstract and concrete categories. *Note.* Dotted lines indicate mean values

At the item level, Fig. 7 shows density plots for the four measures of category production and typicality rating for concrete and abstract categories. There were no clear differences in how frequently member concepts were named for their category (i.e., production frequency) or in how often particular concepts tended to be named *first* for their category (i.e., first-rank frequency). In mean rank, abstract categories had a slightly lower mean than concrete categories (i.e., member concepts tended to be named in earlier ordinal positions), which likely reflects the fact that participants tended to list fewer member concepts for abstract categories. The largest difference occurred in RT for first-named member concepts, where participants were approximately 800 ms slower to produce a response for abstract categories than for concrete categories, implying that abstract category members were more effortful to produce than concrete. Lastly, typicality ratings were slightly higher (i.e., more typical) for abstract than for concrete category members; that is, participants tended to produce “better” examples of abstract categories than of concrete categories. This pattern could arguably be related to category size, whereby the larger concrete categories were more likely to include unusual members than the relatively smaller abstract categories. Very few members of abstract categories were extremely low in typicality (i.e., ≤ 2 on the 1–5 typicality scale; e.g., the lowest rating of 1.83 was for *nine* as a PRIME NUMBER), whereas concrete categories contained several members with low typicality (e.g., rating of 1.17 for *cupboard* as a ROOM IN A HOUSE; or 1.42 for *rabbit* as a RODENT).

**Fig. 7:**
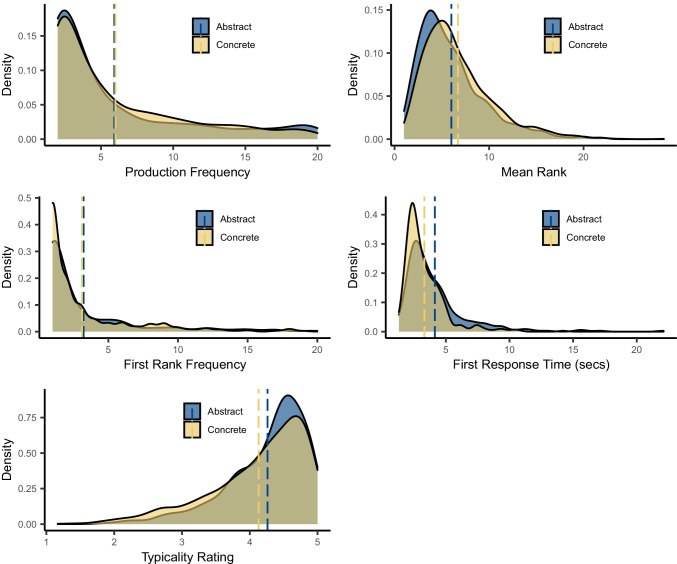
Density plots of category production measures and typicality for abstract and concrete category members. *Note*. Dotted lines indicate mean values. Since all measures exclude idiosyncratic items, the minimum value for production and first-rank frequency is 2. Response times are for first-named category members only

In terms of psycholinguistic variables, the differences between concrete and abstract category members were mostly rather small (see Fig. 8). There was no clear difference in word frequency, but abstract category members were on average one letter longer than concrete members. Members of abstract categories were acquired on average one year later than members of concrete categories, although (as visible in the density plot) they also had a bimodal distribution: many abstract category members were acquired around the age of 5–6 years, similar to the mode of concrete concepts, but a second peak occurred around 7–8 years.

**Fig. 8:**
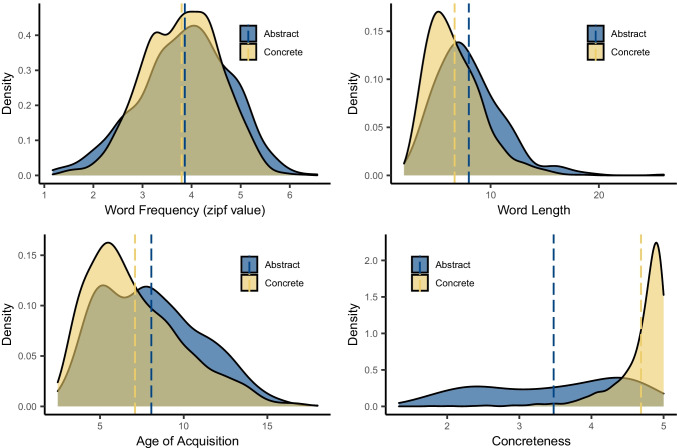
Density plots for psycholinguistic measures of abstract and concrete category members. *Note*. Dotted lines indicate mean values

Unsurprisingly, the two domains greatly differed in concreteness, with abstract category members having a much lower mean concreteness rating than concrete category members. Abstract category members also had a much flatter distribution across the concreteness scale, which meant—somewhat counterintuitively—that many members of abstract categories were actually highly concrete. For example, 13% of abstract category members had ratings of 4.5 or above on the 1–5 concreteness scale (e.g., PROFESSION: *teacher*; SPORT: *ice skating*; GEOMETRIC SHAPE: *rectangle*). Indeed, when the average concreteness of each category was calculated as the mean rating of its constituent member concepts (see Fig. 9), 70% of *abstract* categories contained *concrete* members on average (i.e., the categories had a mean rating above the midpoint of 3 on the 1–5 scale), for example DAY OF THE WEEK, PRIME NUMBER, and SPORT. By comparison, all concrete categories contained concrete members on average, and some 64% of concrete category members were highly concrete (i.e., rated above 4.5). The relative differences in overall concreteness are further illustrated in Fig. 9, which plots all 117 categories ordered by their mean concreteness rating per category. A clear distinction between concrete and abstract categories is apparent but is not as clear-cut as might be expected. Many categories traditionally defined as concrete are indeed at the highest end of the scale (e.g., ANIMAL, FRUIT, VEGETABLE, FURNITURE, TOOL), and many clearly abstract categories are at the lowest end of the scale (e.g., PERSONAL QUALITY, EMOTION, RELIGION, POLITICAL SYSTEM, UNIT OF TIME). Nonetheless, the average concreteness of many abstract categories was still relatively high and comparable to that of concrete categories (e.g., THREE-DIMENSIONAL SHAPE, RACKET SPORT) and, conversely, some concrete categories had average concreteness comparable to many abstract categories (e.g., DRUG, WEATHER). In other words, the abstract nature of a category does not necessarily reflect the abstractness of its member concepts, which may be due at least in part to the role of relations in forming certain categorical groups (e.g., Gentner & Kurtz, 2005; Rehder & Ross, 2001).

**Fig. 9:**
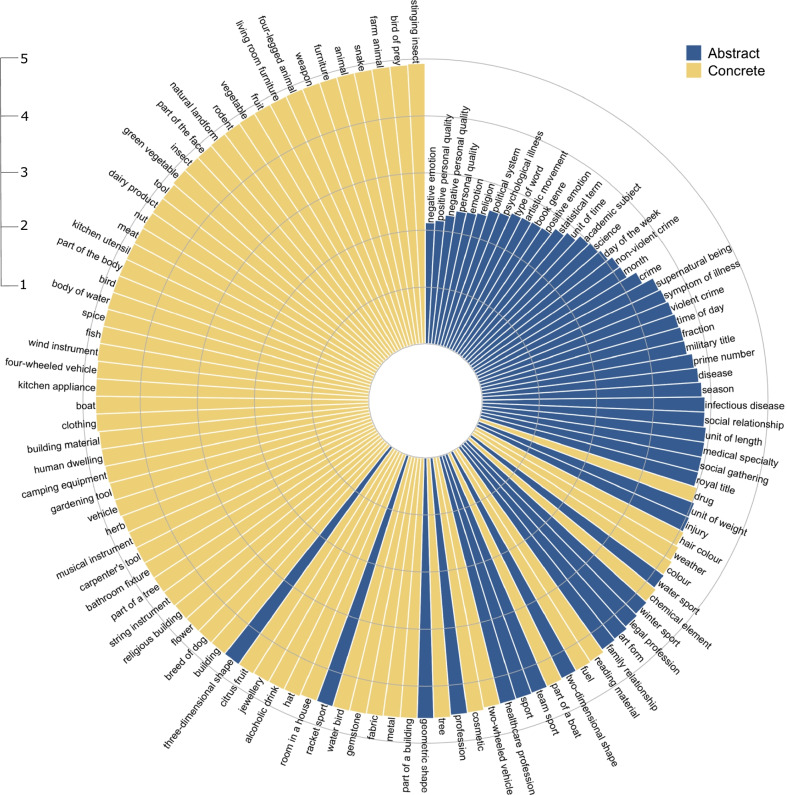
Polar plot showing mean concreteness rating per category (ordered lowest to highest clockwise) for concrete and abstract categories

## Conclusion

We provide the first comprehensive set of UK category production norms for a large number of concrete and abstract categories, including two category-level measures (category size and mean number of responses) and five item-level measures (production frequency, mean rank, first-rank frequency, response times to the first-named members, plus separately normed typicality ratings for most items). In addition, we provide two versions of the norms: a referential version that groups together responses relating to the same core referent, and a full version that retains all lexical variations in responses as produced by participants. We also provide the trial-level data for each participant, including original voice recordings where consent allows, to enable more detailed analyses. These norms represent a timely update and extension of previous category production norms from the UK, which capture important regional differences in category structure compared to contemporary USA, and generational differences within the UK over the last 35 years. Finally, the norms incorporate an extensive set of abstract categories; we provide the first comparison of category production norms for concrete and abstract categories, highlighting structural and psycholinguistic differences between them, and observing that the constituent members of abstract categories can in fact be highly concrete. We hope that the norms and analyses will be of interest and use to a broad range of researchers in cognitive psychology, neuropsychology, psycholinguistics, cognitive modelling, and any field interested in semantic category structure or the process of producing category members.

## Appendix

The variables included in category production norms are as follows.

### Category-level variables

A. *Category* = category name presented to participants
B. *Domain* = whether category was abstract or concrete
C. *category.size* = number of unique member concepts (produced by more than one participant) that were named for a given category
D. *mean.number.responses* = average number of responses produced by each participant per category (total number of non-idiosyncratic participant responses for the category divided by the total number of participants)
E. *category.openness* = extent to which category responses were closed (i.e., each participant listed the same, fixed set of members) or open (i.e., each participant listed a completely different set of members); 1-(mean number responses/category size)
F. *idiosyncratic.members* = number of idiosyncratic category members produced for the category
G. *mean.typicality* = mean typicality rating for the category
H. *mean.wordfreq.SUBTLEXUK =* mean zipf frequency of all category members
I. *mean.word.length* = mean word length (number of letters) of all category members
J. *mean.AoA.Kuperman* = mean age of acquisition of all category members
K. *mean.concreteness.Brysbaert* = mean concreteness rating of all category members

### Item-level variables

A. *category* = category name presented to participants
B. *category.member* = member concept produced by participants for that category
C. *domain* = whether category was abstract or concrete
D. *prod.freq* = production frequency: number of participants who named a particular member concept within its category
E. *prod.freq.percent* = production frequency expressed as a percentage (i.e., divided by total number of participants)
F. *mean.rank* = mean ordinal position of a particular member concept within its category
G. *first.rank.freq* = number of participants who named a particular member concept *first* within its category
H. *first.rank.freq.percent* = first-rank frequency expressed as a percentage (i.e., divided by total number of participants)
I. *weighted.rank = *the production frequency of a given member concept in its category weighted by the ordinal rank position in which each individual participant named it
J. *mean.RT* = mean response time (across all participants) of the category member when named as a first response
K. *typicality* = typicality rating
L. *wordfreq.SUBTLEXUK =* zipf frequency
M. *word.length* = number of letters
N. *AoA.Kuperman* = age of acquisition
O. *concreteness.Brysbaert* = concreteness rating

## Footnotes

1 To maximise clarity throughout the paper, we use uppercase for category names and lowercase italics for member concepts: for example, the category ANIMAL contains the members *cat*, *dog*, and *elephant*.

2 Item-level RTs correlated weakly with lexical variables relating to the presented category name (word length in letters, *r* = .22, < 5% shared variance; number of syllables, *r* = .22, < 5%; and log word frequency, *r* = .02, < 1%); that is, first-response RTs were not confounded with lexical properties of the category name. These analyses are available on the OSF as supplemental material at https://osf.io/jgcu6/.

3 In the USA, *cider* is typically non-alcoholic while *hard cider* is alcoholic; nonetheless, this geographic difference between norms is cultural rather than terminological, because both *cider* and *hard cider* are absent from the US norms for ALCOHOLIC DRINK.
